# Optimizing the quality of horticultural crop: insights into pre-harvest practices in controlled environment agriculture

**DOI:** 10.3389/fpls.2024.1427471

**Published:** 2024-07-23

**Authors:** Xinyi Zhao, Jie Peng, Li Zhang, Xiao Yang, Yujie Qiu, Chengcheng Cai, Jiangtao Hu, Tao Huang, Ying Liang, Zonggeng Li, Mengliang Tian, Fan Liu, Zheng Wang

**Affiliations:** ^1^ College of Agronomy, Sichuan Agricultural University, Chengdu, China; ^2^ Institute of Urban Agriculture, Chinese Academy of Agriculture Sciences, Chengdu, China; ^3^ Vegetable Germplasm Innovation and Variety Improvement Key Laboratory of Sichuan Province, Horticulture Research Institute, Sichuan Academy of Agricultural Sciences, Chengdu, China

**Keywords:** controlled environment agriculture, pre-harvest practices, nutrient supply, light regulation, plant-growth regulator, horticultural crop

## Abstract

In modern agriculture, Controlled environment agriculture (CEA) stands out as a contemporary production mode that leverages precise control over environmental conditions such as nutrient, temperature, light, and other factors to achieve efficient and high-quality agricultural production. Numerous studies have demonstrated the efficacy of manipulating these environmental factors in the short period before harvest to enhance crop yield and quality in CEA. This comprehensive review aims to provide insight into various pre-harvest practices employed in CEA, including nutrient deprivation, nutrient supply, manipulation of the light environment, and the application of exogenous hormones, with the objective of improving yield and quality in horticultural crops. Additionally, we propose an intelligent pre-harvest management system to cultivate high-quality horticultural crops. This system integrates sensor technology, data analysis, and intelligent control, enabling the customization of specific pre-harvest strategies based on producers’ requirements. The envisioned pre-harvest intelligent system holds the potential to enhance crop quality, increase yield, reduce resource wastage, and offer innovative ideas and technical support for the sustainable development of CEA.

## Introduction

The global population is projected to continue growing over the next three decades, with the United Nations estimating it will reach 9.7 billion by 2050 (UN, 2024a). Meeting the increasing food demand poses a primary challenge for modern horticulture, compounded by the challenges presented by climate change for vegetable growers (UN, 2023; UN, 2024b). To address these issues, controlled environment agriculture (CEA) is widely used as a sustainable, high-yield, high-quality production system, offering numerous advantages over traditional agricultural methods. By exercising precise control over environmental factors, light, nutrients, and temperature, CEA significantly enhances crop yield and nutrient quality, irrespective of the location or prevailing weather conditions (Gómez et al., 2019). Over the past two decades, there has been a growing demand for high-quality horticultural products from both consumers and regulatory bodies, emphasizing freshness, nutrition, and functionality (Kyriacou et al., 2016; Zhang et al., 2020). Recent reviews have highlighted the complex compositional aspects of horticultural crop quality, encompassing organoleptic, nutritional, and bioactive components (Kyriacou and Rouphael, 2018).

Horticultural crops are rich in minerals, such as iron (Fe), zinc (Zn), selenium (Se), and iodine (I), as well as phytochemicals including pigments and bioactive compounds (Das et al., 2018). These health promoters constitute a significant portion of the nutrient intake derived from dietary horticultural crops, which provide viable solutions for improving human health through long-term dietary interventions. Improving the nutritional quality and bioactive compounds of horticultural crops has been shown to effectively improve the overall notional status of people (Das et al., 2018; Renna et al., 2022). However, the nutritional quality of horticultural crops can vary widely due to different environmental conditions (e.g., light, temperature, nutrient supply) and agronomic practices (Proietti et al., 2021).

Several pre-harvest practices have been reported to improve crop quality and increase nutrient value by manipulating environmental factors such as nutrient supply, supplemental light condition, and exogenous hormone application. These practices aim to improve nutrient level and taste in crops by optimizing nutrient absorption, metabolism, and accumulation. In this review, we aim to summarize the literatures on various agronomic practices used as pre-harvest strategies to improve horticultural crop quality in CEA systems. These practices include regulating nutrient supply, mediating light environmental conditions such as light period, light intensity, and light quality, as well as applying exogenous plant hormones. By exploring the effects of these practices, we can gain insights into how they enhance crop qualities in CEA systems.

## Agronomic practices

### Nutrient supply

Malnutrition is a worldwide issue affecting the health of billions of people (Gómez et al., 2019), with its prevalence exacerbated by the emergence of COVID-19 (Mark et al., 2020). It is primarily caused by inadequate, unbalanced, or excessive intake of macronutrients (e.g., calcium, potassium, phosphorus) and/or micronutrients (e.g., iron, selenium, iodine). Malnutrition not only directly threatens health but also weakens the body’s defenses, increasing the risk of diseases such as angular cheilitis, pneumonia, anemia, and metabolic disorders (WHO, 2022). Nutrition intake through food is a core of any strategy to reduce and alleviate the global burden of disease (Graham et al., 2007). It is important to note that agricultural products serve as the primary source of nutrients for human beings (Das et al., 2018). Therefore, optimizing the nutritional composition of these products in our diet has the potential to significantly enhance human health. Individuals affected by specific diseases may require an increased consumption of particular nutrients compared to the standard dietary reference (DRV). This personalized approach to nutrition is commonly referred to as personalized nutrition (Renna et al., 2022). For example, chronic kidney disease needs patients to intake low-potassium personalized vegetables in their diet (D’Imperio et al., 2019; Levine and Mattson, 2021). Eating boron (B) biofortified vegetables contributes to bone health and alleviates osteoporosis. Se supplementation by eating Se-fortified vegetables can alleviate the symptoms of Keshan disease, interfere with the expression of key inflammatory genes to prevent tissue damage (Harper et al., 2018; Sasson et al., 2021). From the perspective of clinical and quality of life, the idea of using some foods with specific nutritional characteristics for personalized matching is beneficial (Renna et al., 2022). Given the increasing awareness of health and healthcare, there is a heightened interest among people in consuming agricultural products that offer high nutrient quality.

Soilless cultivation is mainly used in CEA, especially through the use of a smart fertigation system. This system allows for precise manipulation of the composition of the nutrient solution, regulating nutrient uptake at each stage of plant development. As a result, it is possible to increase the concentration of beneficial elements while reducing the presence of elements harmful to human consumption. At present, pre-harvest practice by changing the specific nutrient element has been widely used in CEA to increase the nutrient quality of the crop. In this part, we summarized the current agronomic practices by manipulating nutrient supply before vegetable harvest and explored how it affects the composition of nutrients and bioactive compounds.

### Nutrient depletion

#### Nitrogen

Nitrogen (N) is an essential element for plant growth and development as it’s a fundamental component of crucial substances in plants, including proteins, nucleic acids and hormones in plants (Vidal et al., 2020). In the soilless culture system, nitrogen was supplied mainly in the form of nitrate, which can lead to excessive accumulation of nitrate during vegetable growth and development. This excess nitrate can then be stored in shoot tissues, posing a potential risk to human health (Wu et al., 2021). Additionally, excessive nitrate supply in the nutrient solution can negatively affect the content of various bioactive compounds, such as ascorbic acid content (Becker et al., 2013) and phenolic compounds (Zhou et al., 2018).

Restricting nitrogen supply for a short duration before crop harvest is widely considered as an effective approach to reduce nitrate content, particularly in leafy vegetables grown hydroponically. Previous studies have shown that by depriving lettuce of nitrogen for 2-4 days during the final growth stage in hydroponic culture can reduce leaf nitrate levels by 29% without affecting fresh biomass yield (Tabaglio et al., 2020). Further investigations revealed that restricting nitrogen supply to levels of 1 mM and 0.5 mM reduced leaf nitrate content by 61.2% and 81.9%, respectively, while complete deprivation resulted in a loss of 84% of nitrate content (Zhou et al., 2018).

In addition to its noticeable impact on reducing nitrate content, pre-harvest nitrogen limitation exerts differential effects on plant secondary metabolites. Withholding the nitrogen source in the nutrient solution for 7 days prior to harvest resulted in a marked increase in the concentrations of phenolic compounds, flavonoids, and anthocyanins in lettuce (Zhou et al., 2018). Similar responses were observed in tomatoes, leading to significant enhancements in antioxidant activity and antibacterial properties (Løvdal et al., 2010). Furthermore, nitrogen limitation resulted in a rapid and effective increase in the levels of vitamin C and glutathione in plants (Zhou et al., 2018).

Short-term nitrogen deprivation prior to harvest results in the efflux of nitrate from the vacuole into the cytoplasm where they it is metabolized. This efflux is coupled with the entry of other ions, which helps to regulate the osmotic potential of cells and subsequently reduces the nitrate content in plant tissues (Liu and Shelp, 1996). However, in the case of plants, particularly leafy vegetables in hydroponics, the nitrogen reserves in leaf tissues are typically abundant enough to sustain growth for a short duration (Liang et al., 2022; Yang et al., 2023). Moreover, nitrogen functions as a signaling molecule to induce multiple transcription factors involved in both primary and secondary metabolic processes in plants (Nemie-Feyissa et al., 2014; Gaudinier et al., 2018). Under nitrogen stress, plants respond by upregulating the activity of antioxidant enzymes and increasing the production of secondary metabolites. These adaptations serve to scavenge reactive oxygen species (ROS) and counteract the oxidative stress induced by nitrogen deficiency (Grassmann et al., 2002). Nitrogen deprivation significantly upregulated the transcription levels of structural genes involved in the synthesis pathways of phenols and flavonoids (Løvdal et al., 2010; Zhou et al., 2018). Notably, the expression of phenylalanine ammonia lyase (PAL), chalcone synthase (CHS), flavonol 3-hydroxylase (F3H), and flavonol synthase (FLS) was prominently increased (Zhou et al., 2018).

#### Potassium

Chronic kidney disease (CKD) is a prevalent disease with a significant impact on public health, attracting considerable attention from researchers. Epidemiological evidence indicates that approximately 10% of adults worldwide are affected by CKD (Tonelli et al., 2006). Disturbingly, projections indicate that kidney disease will be the fifth leading cause of death worldwide by 2040 (Tonelli et al., 2006). Patients with CKD often experience potassium (K) accumulation in their bodies due to impaired renal function, potentially leading to complications such as hyperkalemia (Tonelli et al., 2006; D’Imperio et al., 2019). Therefore, it is important for patients to limit their intake of K-rich vegetables (Tonelli et al., 2006).

In conventional soilless culture systems, the nutrient solution often contains elevated levels of K. Therefore, numerous studies have aimed to reduce the K content of vegetables by lowering the of K^+^ concentration in the nutrient solution during the pre-harvest stage. For example, when the K content of the nutrient solution was adjusted from 200 mg·L^-1^ to 50 mg·L^-1^ 7 days before harvest, the K content of spinach decreased by 6.8%, and that of Swiss beet decreased by 15.6%. Moreover, the content of calcium (Ca) and magnesium (Mg) in Swiss beet increased after K stress (D’Imperio et al., 2019). It is worth noting that with the decrease of K concentration, the yield of fresh weight and dry weight also decreased. In a 14 d experiment limiting K-limited experiment of spinach, compared with 100% K treatment, the K content in spinach leaves decreased by 91% and the fresh weight decreased by 76% after 0% K treatment. This suggests that it is possible to reduce K in spinach plants, further research is needed to determine the optimal parameters required to minimize yield loss by limiting K to the greatest extent (Levine and Mattson, 2021). These fundings demonstrate the feasibility of utilizing nutrient solution deprivation in hydroponic vegetable production to finely regulate the supply of K, thereby enabling the development of tailored nutrition strategies for patients with CKD. By employing this approach, CKD patients can increase their intake of low K vegetables without the risk of excessive K intake. Consequently, it enables the utilization of minerals and bioactive substances that have beneficial effects on health.

In conclusion, nitrogen stress before harvest has emerged as a well-established technique for reducing nitrate content and enhancing nutrient qualities, including ascorbic acid, flavonoids, anthocyanins and phenols, particularly in hydroponic leaf vegetable. However, the trade-off between yield reduction and qualities improvement varies among different vegetable varieties. Additionally, the application of nitrogen limitation in fruit vegetable production remains limited due to the challenge of determining the optimal processing time, especially considering that fruit vegetables are harvested multiple times during their growth stage. Therefore, it is challenging for growers to utilize these practices effectively to produce the high-quality vegetables in soilless cultivation system. Future studies should focus on optimal nitrogen limitation processing time and nitrogen limitation dose to strike a balance between vegetable yield and accumulation of bioactivity compounds in both leaf and fruit vegetable, ultimately providing systematic strategies for producers.

In comparison to nitrogen, the effect of K limitation at pre-harvest on primary and secondary metabolism in horticultural crops was still unclear. Future research efforts should aim to elucidate the regulatory mechanisms of K stress on plant growth and metabolism process, reveal the molecular mechanism underlying its influence on yield and quality, and provide practical insights for producers in conjunction with the production practices of different crops.

### Nutrient enrichment

Personalized nutrition, defined as “tailoring dietary recommendations to specific biological requirements based on a person’s health status and goals” (van Ommen et al., 2017), involves customizing dietary advice to meet individual needs. In contrast, biofortification offers an efficient approach to enhance the nutrient quality of leaf and fruit vegetables during the growth phase, and has shown promise in the field of personalized nutrition (Renna et al., 2022). Indeed, biofortification has already been successfully applied to the production of mineral-fortified vegetables. Soilless cultivation presents an opportunity to precisely regulate the nutrient solution to enhance the nutrient uptake and accumulation of nutrients in the edible parts of vegetables (Rouphael and Kyriacou, 2018). This section aims to provide an overview of the current pre-harvest practices involved in hydroponics for cultivating biofortified vegetables.

#### B biofortification

Boron (B) is not traditionally considered an essential nutrient for humans, but evidence suggests that B may improve Ca absorption and vitamin D utilization efficiency, potentially benefiting bone health (Hunt, 2012). B deficiency can pose a risk to human health, increasing the likelihood of diseases such as chronic bone disease (Stone, 2009). The consumption of biofortified vegetable rich in B could offer advantageous effects on bone health, particularly for individuals adhering to personalized dietary regimens, including those diagnosed with osteoporosis.

Previous research has utilized the floating hydroponic cultivation method to increase the concentration of B in the nutrient solution, promoting various degrees of biofortification. For instance, during the purslane harvest period (30 days after sowing), increasing the B concentration from 0.3 mg·L^-1^ to 6 mg·L^-1^ result in a significant elevation of B concentration increased by 1.8-fold and 10.7-fold, respectively. Additionally, the Fe content increased significantly, while the nitrate content decreased (D’Imperio et al., 2020). Similar results were observed in two varieties of basil (purple and green leaf), where a10-fold increase in B concentration in the nutrient solution (from 0.2 mg·L^-1^ B as control) over a 20-day duration resulted in a 40% and 14% increase in shoot B concentration, respectively (Landi et al., 2013). Moreover, the heightened B concentration exhibited significant varietal differences in improving antioxidant capacity. However, when the B concentration was increased 100-fold, clear toxic symptoms were observed (Landi et al., 2013). Above results indicated the importance of considering various factors, such as the crop variety, B application rate and the processing time, when employing the strategy of B biofortification in hydroponic system.

#### Se biofortification

Selenium (Se) is an essential mineral critical for several metabolic pathways in the human body (Wichman et al., 2016). However, inadequate intake often results in insufficient expression of selenium protective proteins, leading to an increased risk of cancer and various chronic diseases such as Keshan and Kashin-Beck diseases (Rayman, 2005; Zhang et al., 2019). Conversely, excessive Se intake can cause toxicity, known as Se disease or alkalosis (Fryer, 2000; Rayman et al., 2018). Seleno-amino acids are widely regarded as an ideal forms for Se biofortification due to their lower toxicity compared to inorganic Se. Human daily consumption of organic Se is approximately 260 μg, whereas inorganic Se intake is only around 16 μg per day (Vinceti et al., 2017).

Adding Se to the nutrient solution effectively increases the Se accumulation in plant tissues. Several factors influence Se biofortification, including the type of Se (inorganic or organic form), application method (foliar or root), treatment timing, and crop species (Hu et al., 2022). Inorganic Se forms like selenite and selenate are commonly used in crop Se biofortification. Plants absorb inorganic Se through the roots and convert it into seleno-amino acids, although with low efficiency and varietal specificity (Yu et al., 2019; Hu et al., 2022). However, supplying high Se concentration through root application can inhibit plant growth and pose potential environmental risks (Tan et al., 2016). Therefore, the appropriate Se application approach must be explored at adaptive rates and time based on different plants selenium tolerance limits to meet dietary requirements for individuals with inadequate intake. Numerous studies have aimed to determine optimal synthesis application strategy for Se, including identifying suitable Se forms, application rates, and timings for various vegetables during the pre-harvest stage. For instance, Puccinelli et al. (2021b) conducted a root application of selenate at a concentration of 13 μM (equivalent to 1 mg Se L^-1^) for a 10-day treatment, resulting in a significant increase in Se content in lettuce leaves from 0.09 mg·kg^-1^ DW to 4.84 mg·kg^-1^ DW. Additionally, Yu et al. (2019) demonstrated that in pak choi (*Brassica rapa*), Se addition significantly increased total Se content in the shoots treated with selenite and selenate, with increases of 8.15 and 3.35 times, respectively, after 24 to 168 hours of Se application. However, few studies have focused on approaches to increase the organic Se levels in vegetables. Further research is still needed in this area (Hu et al., 2022).

#### I biofortification

Iodine (I) is an essential trace element for maintaining human health. Inadequate I intake can lead to I deficiency disorders (IDDs), including diseases such as goiter, dementia, reduced IQ, and conditions such as cretinism (Li et al., 2017a; Incrocci et al., 2019). Despite systematic salt iodization in the market, certain individuals still face the risk of I deficiency due to its volatility and varying intake practices. As early as 2008, scholars highlighted that fortified vegetables contain highly absorbable I, with the human body being able to uptake up to 99% of the total amount (Weng et al., 2008). Consequently, biofortification of vegetables has gained prominence as a potential solution.

Similar to Se, biofortification of vegetables with I should consider the potential effects of both deficiency and excess on human health and plant growth. The World Health Organization (WHO) recommends a daily I intake range of 150-300 μg for adults (2017 World Health Organization). Remarkably, fortified vegetables provide I amounts that exceed this daily requirement, effectively meeting the recommended intake levels. Short-term pre-harvest I biofortification can effectively increase the I-content in plant tissues. For instance, the I level in sweet basil and lettuce leaves after 7-14 days of pre-harvest I biofortification ranged from 9760-23580 μg·kg^-1^ and 1550-3600 μg·kg^-1^, respectively (Puccinelli et al., 2021a). I biofortification positively affects plant quality and antioxidant capacity. Supplementation of 0.25 mg·L^-1^ I^-^ in nutrient solution increased the content of vitamin C and soluble sugars in strawberry fruits (Li et al., 2017a). In lettuce, biofortification with 10 μM iodine increased the phenolic compounds in leaves by 44%, 2,2-Diphenyl-1-picrylhydrazyl (DPPH) free radical scavenging activity by 26.3%, and fluorescence recovery after photobleaching (FRAP) by 20.5% (Puccinelli et al., 2021a). Furthermore, the concentration and ion form of I (i.e. I^-^ and IO_3_^-^) plays a crucial role in I bioaugmentation, directly affecting plant growth and I content accumulation. For example, after 14 days of incubation in nutrient solution containing 200 μM KI, the I content in lettuce leaves enhanced by IO_3_^-^ (5433 mg·kg^-1^ DW) was significantly higher than that of I^-^ (4250 mg·kg^-1^ DW) (Puccinelli et al., 2021a).

On the other hand, excessive exogenous I has been shown to adversely affect plants by inhibiting crop growth. In iodine biofortification of strawberry, the aboveground biomass per plant was lower than the control when the concentration exceeded 0.5 mg·L^-1^, with biomass decreased with the increasing I concentration (Li et al., 2017a). Moreover, different I ion forms exhibit varying toxicity to plants, with I^-^ > IO_3_^-^ (Incrocci et al., 2019, 2019). In hydroponic cabbage cultivation, 0.1 mg·L^-1^ of iodide led to a reduction in biomass, while 0.5 mg·L^-1^ iodate resulted in a significant biomass decrease (Weng et al., 2008). Incrocci et al. (2019) further confirmed that both I forms and sweet basil varieties influence plant I tolerance in hydroponic systems. Plant growth was unaffected by either 10 μM KI or 100 μM KIO_3_. However, concentrations of KI higher than 50 μM resulted in significant reductions in leaf area, total plant dry matter, and plant height. The severity of symptoms varied with time, depending on the cultivar and the form of I application. Specifically, growth inhibition caused by toxic I concentrations was more severe in the green leaf variety compared to red leaf variety. Furthermore, KI exhibited higher phytotoxicity than KIO_3_ (Zhu et al., 2003). The low toxicity of IO_3_^-^ may be attributed to its requirement for reduction to I^-^ through electrochemical or enzymatic processes before plant absorption occurs (Kato et al., 2013).

In conclusion, increasing specific trace elements levels in the nutrient solution has shown significant enrichment in the edible parts of vegetables, offering benefits for human nutrition. However, when considering the biofortification of elements such as B, Se, and I, it’s crucial to carefully evaluate the selection of enrichment approaches, considering their potential adverse effects on plants and possible implications for human toxicity. Exploring innovative cultivation patterns and production systems, such as hydroponics, vertical farming, or CEA, can offer new avenues for enhancing trace-element biofortification in horticultural crops. These modern cultivation methods allow for precise control over environmental factors, nutrient delivery, and plant growth, thereby facilitating targeted biofortification efforts.

Future studies should continue to optimize pre-harvest strategies concerning trace-element form, application rate, method, processing time, and cultivation patterns for various horticultural crops. These optimizations should be coupled with an exploration of the underlying mechanisms involved. By investigating deeper into these aspects, researchers can refine and tailor biofortification techniques to maximize their effectiveness while minimizing potential risks. Therefore, understanding the mechanisms underlying trace-element uptake, translocation, and metabolism within plants will provide valuable insights into how to optimize biofortification strategies. Additionally, considering the diverse range of horticultural crops and their unique physiological characteristics, it’s crucial to investigate how different species respond to various biofortification approaches. Moreover, there should be increased emphasis on combining various trace elements at pre-harvest stage to develop compound nutrient-rich vegetables, offering a more comprehensive functional food option for specific populations. This approach ensures not only enhanced nutritional benefits but also addresses potential health risks associated with excessive trace element intake. Overall, future studies should focus on comprehensive optimization of pre-harvest strategies for trace-element biofortification in horticultural crops, supported by a thorough understanding of the underlying physiological and biochemical mechanisms. Such endeavors will contribute to the development of sustainable and effective approaches to improve the nutritional quality of crops and address global health challenges related to nutrient deficiencies.

### Light regulation

In the field of CEA, effectively managing the light environment is paramount for maximizing crop production (Bantis and Koukounaras, 2023). The utilization of artificial lighting, such as light-emitting diodes (LEDs), empowers growers with precise control over the photoperiod, light spectrum, and light intensity for their crops (Wu, 2021). In the short term, manipulating the light environment before harvest mainly involves extending the duration of illumination (continuous light, CL) and providing additional light to crops. Notably, ultraviolet radiation plays a pivotal role in the photomorphogenesis of crops (Velez-Ramirez et al., 2011). In this part, we will summarize recent strategies for regulating light in vegetable during the pre-harvest period, with the objective of optimizing both yield and quality of horticultural crop by manipulating the light environment.

#### Continuous light

CL refers to the practice of providing plants with uninterrupted illumination for a specific duration, thereby disrupting the natural light and dark photoperiod established by the plant’s circadian rhythm (Velez-Ramirez et al., 2011). CL breaks the original circadian rhythm of plants and is a kind of light stress for plants. It prolongs the time of photosynthesis of plants, and many plants increase dry matter accumulation under CL (Murakami et al., 2011). Implemented as a pre-harvest strategy in agricultural production, CL aims to manipulate plant growth and development with the ultimate objective of improving yield and quality. The primary goal of employing CL as a pre-harvest strategy is to increase crop productivity by stimulating photosynthesis and optimizing various physiological processes. CL exposure has been observed to enhance overall photosynthetic activity, leading to improved carbohydrate accumulation in plant tissues in a short period (Bian et al., 2018). However, it’s important to note that CL can induce stress condition, potentially leading to adverse effects such as disturbed circadian rhythm, heightened susceptibility to photoinhibition and oxidative damage, and altered hormonal regulation (Velez-Ramirez et al., 2011). In practice, numerous studies have focused on optimizing the light duration, spectrum and intensity of CL to achieve optimal yields and nutrient quality in horticultural crops (Bian et al., 2018; Proietti et al., 2021; Lanoue et al., 2022). These investigations aim to strike a balance between maximizing the beneficial effects of CL on plant growth, metabolism and mitigating its potential negative impacts on plant development and productivity.

##### Light duration

CL applied for a short period before harvest has been shown to increase the biomass of plants. Bian et al. (2016) reported that after 72 hours of CL treatment, lettuce fresh weight increased by 19.6%, dry weight by 76.3%, and leaf area by 46.3%. Similar results have been observed in medicinal plants and fruits and vegetables such as chicory, sweet pepper, and tomatoes (Gislerød et al., 1989; Ohyama et al., 2005; Xu et al., 2021). Additionally, Liu and Liu, 2022 found that CL for 12 days significantly increased the starch and sucrose content in vegetables. These findings are attributed to the extended duration of light exposure, which increases leaf area and chlorophyll content, thereby enhancing photosynthetic efficiency, improving the accumulation of photosynthetic products, and ultimately increasing plant biomass. However, extending CL to 21 days can lead to leaf chlorosis in cucumber plants, a significant decrease in starch content, and a decline in yield (Pettersen et al., 2010). This may be due to the excessive duration of CL causing photosynthesis to exceed its capacity, resulting in the inhibition of electron transfer in the photosynthetic redox reaction chain, and ultimately causing photooxidative damage (Pettersen et al., 2010). In addition, the varying sensitivity of crops to light leads to differences in their performance under CL. Under the same conditions, CL decreased the leaf area mass (LMA) of tomato leaves but had no effect on that of cucumber leaves (Shibaeva et al., 2022). CL reduced the chlorophyll content of eggplant and tomato by 40-80%, but the chlorophyll content of sesame cabbage under the same conditions was 18% higher than that of the control plants (Shibaeva et al., 2023). The difference in plant photosensitivity also led to variations in metabolite accumulation under CL. For example, CL increased the content of phenolics and anthocyanins and enhanced antioxidant activity in amaranth and kale, but the biochemical components of green basil and violet basil remained unchanged under the same conditions (Lanoue et al., 2022). This variation is related to the crop species and their sensitivity to light exposure, which should be fully considered in the application of pre-harvest light system.

Certain primary and secondary metabolites in plants also undergo corresponding changes under CL conditions. For instance, in hydroponic lettuce, CL treatment for 48 hours significantly reduces nitrate content (Zhou et al., 2015). One possible explanation for these changes is that CL significantly enhances the accumulation of ELONGATED HYPOCOTYL5 (HY5), leading to increased activity of nitrate reductase (NR) (Osterlund et al., 2000; Jonassen et al., 2009). This increased activity is associated with the upregulation of NR-related genes in plants (Jonassen et al., 2009). Gradually extending CL to 72 hours, compared to 48 hours, may result in an increasing trend in nitrate content (Bian et al., 2016). This trend could be attributed to the significant increase in the activity of the key rate-limiting enzyme NR in the nitrate metabolism pathway after 48 hours of CL, as the CL exposure time increases, changes in nitrite reductase (NiR) and glutamine synthetase (Gs) lead to a drastic decrease in NR activity, resulting in a trend of initial decline followed by increase (Osterlund et al., 2000; Jonassen et al., 2009; Bian et al., 2018). Vitamin C, also known as ascorbic acid (AsA), plays a crucial role as an antioxidant in plants. CL treatment for 72 hours has been shown to enhance the accumulation of AsA and dehydroascorbic acid (DHA) (Bian et al., 2016; Zhang et al., 2021). This enhancement is attributed to the increased activity of l-galactose-1,4-lactone dehydrogenase (GalLDH), involved in AsA synthesis, and dehydroascorbate reductase (DHAR), involved in the recycling of DHA to AsA (Bian et al., 2016; Zha et al., 2019b).

In conclusion, the approach of extending the duration of the light cycle during the harvest stages of horticultural crops have proven effective in reducing nitrate content and increasing the levels of AsA and other bioactive compounds. This results in the production of vegetables that offer significant health benefits to humans.

##### Light quality

Light wavelength is an important environmental factor that affects plant growth, as well as primary and secondary metabolism (Pierik and Ballare, 2021; Liang et al., 2023). Plants detect and absorb red and blue light spectra through photoreceptors to facilitate photosynthesis in chlorophyll, such as phytochromes, which detect red and far-red light, and cryptochromes, which detect blue light (McCree, 1971). The activation of these photoreceptors induces light-dependent metabolic changes and regulates essential physiological processes for plant growth, development, and environmental adaptation (Li and Kubota, 2009; Demotes-Mainard et al., 2016; Huché-Thélier et al., 2016). Compared to continuous white light, the implementation of continuous red and blue combined light significantly increased lettuce yield, with fresh weight and dry weight improvements of 32.1% and 46.1%, respectively. These values were also notably higher compared to lettuce that did not receive CL treatment, which showed an increase of 58% and 119%, respectively (Bian et al., 2016). Notably, there was a positive correlation observed between the higher ratio of red light under CL and the increase in plant biomass. Previous study suggests that CL with a red-blue light quality ratio of 4:1 is more conducive to increasing the biomass in lettuce (Zhang et al., 2021). Furthermore, altering the composition of different light wavelengths has a significant effect on important nutritional characteristics of plants. Initially, RBG-CL led to a decrease in NO_3_^-^ concentration within 36-60 hours, but this reduction became less pronounced with prolonged exposure. Moreover, the activity of NR was markedly increased by RBG-CL at 60 hours, but subsequently declined, highlighting the circadian rhythm associated with the decrease in nitrate content (Bian et al., 2016). In comparison to white light, CL also led to an increase in the AsA content when exposed to a red and blue light ratio of 4:1. This elevation is attributed to the heightened activity of key enzymes involved in the AsA synthesis pathway, including L-galactono-1,4-lactone dehydrogenase (GAILDH), ascorbate peroxidase (APX), monodehydroascorbate reductase (MDHAR), and dehydroascorbate reductase (DHAR) (Zhang et al., 2021). Therefore, considering the appropriate light composition in the pre-harvest CL strategy for plants may lead to a more favorable response in the further improving nutritional quality of vegetables.

##### Light intensity

In addition to photoperiod and light quality composition, the intensity of CL also remarkably affects plant growth and quality formation at pre-harvest stage. In a study involving Sesamum indicum, it was observed that CL with medium PPFD (300 μmol m^-2^ s^-1^) was more conducive to increase in both fresh weight and dry weight in plants compared to CL with higher PPFD (600 μmol m^-2^ s^-1^) (Proietti et al., 2021). Previous studies revealed that exposing lettuce to normal light intensity (150-200 μmol m^-2^ s^-1^) during specific time periods, as opposed to low light intensity (50-100 μmol m^-2^ s^-1^), resulted in significant enhancements in soluble sugar and vitamin C content, along with notable reductions in nitrate content (Zhou et al., 2015; Liu et al., 2020). This phenomenon is likely attributed to the elevated synthesis and activity of NR, as well as the enhanced activity of MDHAR and DHAR enzymes under higher light intensity, contributing to increased nitrate consumption and AsA synthesis (Xu et al., 2021). It is well-known that under normal photoperiod, the use of high light intensity irradiation can increase the photosynthetic rate of plants, nitrate assimilation, and other physiological processes, positively influencing plant growth (Min et al., 2021; Darko et al., 2023; Proietti et al., 2023). However, when applying high-intensity light flux in CL, it creates two forms of light stress (photoperiod and light intensity), which may not have a proportional regulatory effect and can even have a negative impact (Zha et al., 2019a).

#### Supplementary lighting

Supplemental light (SL) serves as a pivotal technology to increase horticultural yields (Zheng et al., 2018). Within the realm of CEA, where artificial lighting, notably LED, has seen significant advancements, the choice of light spectrum holds paramount importance in the application of supplementary lighting technology. In this section, we present an overview of the current research landscape concerning supplemental lighting in CEA, highlighting investigations into different light qualities and their implications.

##### Red light, far-red light and blue light

In CEA, growers frequently enhance production efficiency and optimize crop characteristics by supplemental lighting at specific stages of plant growth (Sena et al., 2024). The utilization of red, far-red, and blue light demonstrates diverse patterns in regulating plant growth, development, and primary and secondary metabolism (Wang et al., 2016; Wei et al., 2023). Researches consistently highlights the effectiveness of red, blue, and far-red LEDs as supplemental light sources to stimulate these physiological processes. Within greenhouse production, investigating the combined use of red, far-red, and blue light with natural light has provided insights into its impact on crop growth, final yield, and the development of quality traits during the pre-harvest stage (Hooks et al., 2022; Sale et al., 2023). Recent research emphasizes that the integration of red and blue light not only boosts crop yield but also significantly influences the accumulation of primary and secondary metabolites, crucial for enhancing plant nutritional quality and this underscores the approach as an optimal pre-harvest strategy (Deng et al., 2017).

In the pre-harvest lighting scheme, the selection of a higher ratio of red to blue light as a supplemental light source was aimed at increasing crop yield, improving nutrient quality, and influencing primary metabolic process such as sucrose and nitrate. This approach has been demonstrated to increase crop yield in lettuce (Długosz-Grochowska et al., 2017), tomato and paper (Palmitessa et al., 2020; Yap et al., 2021). Supplementing with red and blue light (R:B=7:2) for 3 h per day for 30 days before harvest significantly increased the activity of Rubisco, facilitated the synthesis and accumulation of photosynthetic pigments, resulting in increased photosynthetic capacity and yield of photosynthetic products (Wang et al., 2022b). Furthermore, a higher proportion of red supplementary light significantly enhances plant sucrose metabolism. Irradiating lamb lettuce with a PPFD of 200 μmol m^-2^ s^-1^ from a 9:1 ratio of red to blue LED light for 16 hours daily over 60 days significantly increased the soluble sugar content, associated with the improvement in the activities of sucrose phosphate synthase (SPS), acid invertase (AI), and neutral invertase (NI) (Borsani et al., 2009; Lombardo et al., 2011; Wojciechowska et al., 2015; Li et al., 2017b). Meanwhile, lettuce supplemented with 8R2B LED light at a PPFD of 200 μmol m^-2^ s^-1^ for 16 h per day for 45 days before harvest significantly reduces nitrate content in leaves by increasing NR activity and accelerating nitrate decomposition (Ohashi et al., 2006). In addition, light supplementation also increased the phenolics (increased by 50%), anthocyanins and antioxidant activity (FRAP increased by 66.7%, DPPH increased by 79.6%) of lettuce. This is attributed to the increased blue light in the combined light, which plays an important role in regulating plant polyphenol synthesis (Długosz-Grochowska et al., 2017). It also should be noticed that blue light strongly induces plant secondary metabolism to improve crop quality. For example, supplementing with blue LED light at intensity ranging from 50 to 100 μmol m^-2^ s^-1^ for 12 hours daily over a 10-day period before harvest significantly increased the content of TPC, TF, TA, TG and antioxidant capacity in Chinese cabbage. This enhancement is attributed to an increase in the catalytic activity of PAL, facilitated by the blue light spectrum, leading to the formation of various phenolic compounds (Zheng et al., 2018).

Moreover, the addition of far-red light irradiation has proven to be a more effective strategy for promoting growth, enhancing crop nutritional content, and ultimately improving quality (Begum et al., 2023). This is particularly evident in the context of improved crop yield and quality observed in a greenhouse environment during the winter season than in the summer (Yan et al., 2023). In a greenhouse trail during the winter season, compared to natural sunlight and supplementary white LED light, the addition of white light combined with far-red light for 25 days resulted in 4 fold increase in lettuce yield. This supplementation also led to a significant elevation in the content of total chlorophyll, carotenoids, soluble protein, Vitamin C, and soluble sugar (Yan et al., 2023). Similar positive outcomes were observed in a study with sweet peppers. When compared to natural light conditions and supplementary red and blue light, the inclusion of supplementary red, blue, and far-red light significantly increased fruit yield, along with sucrose, glucose, fructose, and total soluble sugar content, while decreasing citric acid, malic acid, and shikimic acid, ultimately enhancing fruit quality and taste (Kim et al., 2023). Increasing the proportion of far-red light (i.e., a lower R: FR ratio) resulted in a significant increase in dry matter weight, attributed to the augmentation of leaf area, thereby enhancing light interception and enabling the plant to tolerate low light conditions (Li and Kubota, 2009). Additionally, transcriptomic analysis revealed that supplementary far-red light enhances chilling tolerance by upregulating the expression of genes associated with various pathways linked to cold tolerance, such as carbohydrate metabolism and anthocyanin biosynthesis (Begum et al., 2023).

The implementation of LED supplemental lighting in CEA is of paramount significance in enhancing the value of horticultural crops. In particular, the use of red and blue light supplementation demonstrates its potential to improve photosynthetic efficiency and nutrient uptake, thereby positively impacting crop yield. Additionally, the regulatory impact of LED supplemental lighting on nutrient absorption and metabolic processes contributes to an improvement in overall crop quality, with red light boosting fruit sugar content and color (Ngcobo et al., 2020), while blue light increases specific compounds in leafy vegetables (Długosz-Grochowska et al., 2017; Zheng et al., 2018). Furthermore, LED lights exhibit extended lifespan, lower heat generation, and high spectral output stability, ensuring a stable growth environment without the risk of heat-induced damage. In terms of sustainability, the LED red and blue light supplement system proves efficient in energy utilization, tailored to different growth stages and crop needs, resulting in reduced energy consumption compared to traditional methods. Despite the initial investment, the long-term benefits of energy conservation and enhanced yield position LED supplementary lighting as an economically viable and profitable solution for CEA.

#### UVs supplementary

Short-term exposure of UVs radiation prior to harvest provides a straightforward, swift, and effective method for improving plant nutrient qualities. Numerous studies have focused on the use of UV radiation, including UV-A (320-400 nm), UV-B (280-320 nm), and UV-C (100-280 nm), as pre-harvest lighting of plants. The intervention of these UV wavelengths typically results in the accumulation of phytochemicals that promote health and nutrients crucial to human health. This approach helps ensure that the vegetables produced are better suited to the needs and preferences of consumers.

##### UV-A

Plants absorb UV-induced blue fluorescence and UV-A through their photosynthetic pigments (Verdaguer et al., 2017). Therefore, supplementing crops with UV-A prior to harvest can enhance photosynthesis and positively affect plant biomass and the accumulation of secondary metabolites (Johnson and Day, 2002). Exposing lettuce to UV-A for four nights before harvest resulted in a notable increase in both fresh weight (68.8%) and dry weight (64.8%) (Hernandez et al., 2021). Likewise, exposing tomato plants to UV-A light for 1 hour resulted in a remarkable increase in the number of tomato fruits by 99.4% (Mariz-Ponte et al., 2019). The influence of UV-A on the quality of pre-harvest vegetables is primarily observed in the enhancement of plant pigments, proteins, phenols, and flavonoids. After exposure to UV-A radiation for 3-6 days before harvest, the levels of carotene, anthocyanin, and total phenol in vegetable leaves increased by 11% to 86% compared to the control group (Hooks et al., 2021; Lee et al., 2022). Additionally, UV-A radiation significantly promoted the accumulation of proteins and essential nutrients such as phosphorus (P), potassium (K) and calcium (Ca), in the edible parts of the vegetables when compared to the control group (Hooks et al., 2021; Lee et al., 2022). UV-A regulates the levels of phenolics and flavonoids through its substantial upregulation of PAL enzyme activity and the transcriptional expression of structural genes involved in flavonoid synthesis, inducing the accumulation of flavonoids and phenolic compounds in plants (Zhang et al., 2013; Jeon et al., 2018; Mariz-Ponte et al., 2019; Lee et al., 2022. UV-A irradiation has been observed to enhance not only the nutritional quality of functional vegetables but also the color, aroma, and fragrance of fruit vegetables, such as tomatoes (Mariz-Ponte et al., 2019). The enhancement of sensory perception in fruit and vegetables is crucial for attracting consumers and improving their commercial value.

##### UV-B

UV-B radiation serves as a potent stimulus for plant metabolic reactions, exerting diverse effects on both plant growth and the synthesis of secondary metabolites (Neugart and Schreiner, 2018). However, a recent study indicated that mild daily UV-B radiation (i.e. 15 min a day corresponding to 1.19 kJ m^-2^) for 11 days before harvest significantly changed the hormone levels in both leaves and roots of tomatoes without negatively affecting their growth (Mannucci et al., 2020). Supplemental UV-B radiation at preharvest stage has been shown to enhance the accumulation of flavonoids, carotenoids, and ascorbic acid in lettuce (Assumpção et al., 2019), as well as in tomatoes (Giuntini et al., 2005; Dzakovich et al., 2016). Moreover, exposing broccoli microgreens to UV-B for five days before harvest significantly increased glucoraphanin (GLR), glucoerucin (GLE) and total aliphatic GL content while also maintaining nutritional quality at the harvest stage (Lu et al., 2021. On the other hand, plants irradiated with low doses of UV-B (21.6 kJ m^-2^) had higher levels of antioxidants and glucosinolates, and the damage of plant membranes was lighter, compared with high doses of UV-B radiation (86.4 kJ m^-2^) (Ali et al., 2023).

UV-B radiation has been observed to exert significant effects on the expression of genes involved in various metabolic pathways. Specifically, it modulates the expression of genes associated with the citric acid cycle, facilitating the production and accumulation of photoprotective flavonoids. In blueberries, using UV-B irradiate for 7 hours per day, 7 days before harvest, can increase the anthocyanin content by involving a specific regulatory network, including *VcMYBA1*, *VcMYBPA1* and *VcMYBC2*, which mediates anthocyanin accumulation induced by UV-B (Li et al., 2021). Furthermore, UV-B radiation induces a substantial increase in transcript levels of genes linked to the polyphenol pathway and the biosynthesis of glucosinolates (GLs), notably CYP79F1 and MAM. These molecular alterations contribute to the promotion and maintenance of the accumulation and stability of primary and secondary plant metabolites (Giuntini et al., 2005; Kusano et al., 2011; Dzakovich et al., 2016; Scott et al., 2021). Moreover, it is noteworthy that exposure to UV-B radiation for a specific duration elicits a substantial upregulation in the expression of the E3 ubiquitin ligase CONSTITUTIVELY PHOTOMORPHOGENIC1 (COP1) and the bZIP transcription factor HY5 at 12 hours, however, their expression becomes negligible after 36 hours of continuous UV-B radiation exposure, indicating plants undergo adaptation to UV-B radiation over time, leading to alterations in gene expression patterns (Dzakovich et al., 2016). In both research and production applications, it is crucial to carefully consider the impact of UV-B exposure duration and dosage on vegetables. This attention is vital due to the substantial influence that the duration and dose of UV-B exposure can have on plant responses and outcomes. Consequently, the potential benefits of employing UV-B supplemental light treatment on vegetables before harvest are promising, as they hold the potential to enhance both nutritional quality and storage characteristics.

##### UV-C

UV-C radiation is recognized for its fungicidal effects, making it an effective way to inhibit post-harvest diseases and reduce plant spoilage, thereby extending the shelf life of fruit and vegetables, a technique commonly used in horticultural practices (Urban et al., 2016, 2018). In addition to its fungicidal properties, UV-C treatment has demonstrated the capability to improve the quality of vegetables. Studies have illustrated that UV-C treatment increases the content of total phenolic compounds (TPC) and antioxidant enzymes in lettuce, spinach, and tomato, thereby augmenting their antioxidant capacity (Vàsquez et al., 2017; Esua et al., 2019; Martínez-Sánchez et al., 2019). Short irradiation of UV-C stimulates the activities of antioxidant enzymes such as superoxide dismutase (SOD), catalase (CAT), and ascorbate peroxidase (APX) (Jin et al., 2017). Furthermore, it up-regulates the transcript levels of key enzymes involved in phenolic compound synthesis, such as PAL, CHS, and F3H (Pinto et al., 2016), promoting the accumulation of plant phenolics and ultimately leading to an increase in plant antioxidant activity. Notably, unlike UV-A and UV-B, UV-C radiation demonstrates efficacy in inhibiting bacterial growth; for instance, a dose of 3 kJ m^-2^ of UV-C controls yeast, mycobacteria, and eosinophilic bacteria growth (Martínez-Sánchez et al., 2019). Additionally, UV-C irradiation, beyond its direct inhibitory effects, induces the upregulation of plant defense-related genes, reinforcing plant resistance to specific pathogens like gray mold (Jin et al., 2017; Esua et al., 2019; Zhang and Jiang, 2019; Zhou et al., 2019). These findings highlight the potential of UV-C treatment in improving plant defense mechanisms against pathogens.

With the growing consumer preference for antioxidant-rich vegetables, exploring various methods to enhance the crop’s nutritional value is imperative. CEA offers promising opportunities, with timed and quantified UV radiation emerging as well-developed pre-harvest strategies to improve crop function and nutritional quality in the future. However, there are inherent limitations to the use of UV-A, UV-B, and UV-C for supplemental lighting in CEA. Excessive exposure of plants to UV light results in the oxidation of cell membranes, DNA damage and photosynthesis, resulting in symptoms such as leaf yellowing, dehydration, stomatal closure and tissue damage, ultimately inhibiting plant growth and development (Jansen, 2002; Nawkar et al., 2013; Lee et al., 2014). Moreover, UV light poses potential risks to human health, such as skin and eye irritation, increased risk of skin cancer and immune system suppression (Simonsen et al., 2017; Yi et al., 2018; Dickinson et al., 2021). Therefore, careful regulation of the intensity and duration of UV radiation is essential to prevent undue damage to both plants and humans. Additionally, integrating supplemental light from other spectra is crucial to comprehensively promote plant growth and development while ensuring overall plant health.

In general, studies on pre-harvest light environment regulation, including CL, LED supplemental lighting across the visible spectrum, far-red, and UV light, demonstrate considerable potential to enhance plant growth, development, primary and secondary metabolism. Consequently, these practices contribute to augmenting the nutritional value, flavor, and taste of horticultural crops. However, conducting a comprehensive cost-benefit analysis is imperative in CEA production. Both CL and supplemental LED lighting incur increased energy costs and necessitate significant initial investments and enhanced technical management, thereby escalating economic and labor costs. Nevertheless, the capacity of pre-harvest light environment regulation to yield higher commodity value and income compared to associated costs is often overlooked by researchers. Consequently, a fundamental focus of current research involves devising strategies to mitigate energy demand in pre-harvest light environment regulation and optimize energy utilization efficiency. Moreover, future studies should explore the integration of various light regulation practices to shorten processing time, thereby positively impacting crop production efficiency and increasing the content of secondary metabolites, ultimately enhancing the characteristics of plant commodities. Further in-depth research and refinement of light parameter optimization and energy consumption reduction are imperative for achieving sustainable development in future agricultural production.

### Plant growth regulator

Plant growth regulators (PGRs) play a pivotal role in vegetable production, extensively utilized to increase yield, enhance secondary metabolic content, prolong shelf life, and elevate commodity value (El-Zaeddi et al., 2017; Miceli et al., 2019; Wang et al., 2021a; Kamakshi et al., 2023). These synthetic compounds or naturally derived extracts mimic the effects of plant hormones, providing several advantages, including low dosage requirements, broad effectiveness, high efficiency, and minimal residue. Commonly employed PGRs for crop harvesting encompass oxalic acid (OA), salicylic acid (SA), abscisic acid (ABA), methyl jasmonate (MeJA), and gibberellin (GA_3_). In this section, we will summarize the current pre-harvest practices using different PRGs to improve horticultural crop yield and nutrient qualities.

#### Oxalic acid

Oxalic acid (OA), recognized as a natural and environmentally friendly compound, has garnered significant research attention in recent years as a pre-harvest strategy to delay post-harvest senescence and preserve crop quality (Del Rio et al., 2013; Martínez-Esplá et al., 2017). Exogenous application of OA three times before harvest (30 L of 2 mM OA applied at 45, 24 and 3 days before harvest, respectively) did not significantly affect the overall yield of artichokes, but significantly increased the proportion and firmness of first-class artichokes while reducing the rate of weight loss, effectively delaying senescence during post-harvest storage. Additionally, OA treatment significantly enhanced the antioxidant capacity and phenolic content, including total hydroxycinnamic acid and total luteolin, in artichokes (Martínez-Esplá et al., 2017) and coriander (El-Zaeddi et al., 2017). The optimization of OA treatment to enhance crop antioxidant activity and phenolic content is primarily attributed to its ability to augment the activity of various enzymes including polyphenol oxidase, as well as key antioxidant enzymes, such as SOD, CAT and APX (Martínez-Esplá et al., 2014; Razavi and Hajilou, 2016; Martínez-Esplá et al., 2017). Therefore, the use of OA treatment in the pre-harvest preparation of functional vegetables holds great importance for individuals with specific health requirements.

#### Salicylic acid

Salicylic acid (SA), recognized as a growth regulator that induces plant disease resistance and improves plant nutritional quality (Li et al., 2022), is effectively employed in pre-harvest strategy in horticultural crop production. For example, the pre-harvest application of 150 μM SA by spraying significantly increased both fresh weight (by 58%) and dry weight (by 50%) of leek plants and enhanced the presence of flavor compounds such as enzymatic pyruvate, sulfide and aromatic volatile compounds (Wang et al., 2021a). Additionally, the spraying of SA has been found to significantly enhance the accumulation of pigments and antioxidant activity such as chlorophyll, phenols, flavonoids, DPPH free radical scavenging activity, 2, 2’-azino-bis (3-ethylbenzothiazoline-6-sulfonic acid) (ABTS) and FRAP, while reducing the nitrate content in leaf vegetables, thereby improving their commercial value (Sun et al., 2012; Wang et al., 2021a). Furthermore, SA application positively influenced plant immunity and post-harvest preservation, significantly inhibiting the incidence of tomato gray mold and delaying fruit ripening by reducing lycopene and ethylene content (Wang et al., 2011). SA’ efficacy in inducing fruit disease resistance is attributed to its ability to mediate the expression of plant disease resistance-related genes (PRs) through exogenous application (Wang et al., 2011).

#### Abscisic acid

Abscisic acid (ABA), an isoprenoid phytohormone, acts as a signaling factor to regulate plant growth, development and stress (Zhao et al., 2005). Treatment with 100 µM ABA did not affect the sensory quality of lettuce, but improved its health compounds including flavonoids, caffeic acid, chlorogenic acid, chlorophyll A and carotene (Złotek et al., 2014). Exogenous application of ABA has been demonstrated to enhance plant adaptability to various abiotic stresses, including cold, drought, and salinity (Sah et al., 2016). ABA functions by regulating stomatal aperture to minimize water loss during drought conditions and inducing the expression of genes involved in stress response and tolerance mechanisms (Złotek et al., 2014). Seki et al. (2002) identified a total of 245 ABA-inducible genes in Arabidopsis, with drought-inducible genes accounting for 63% (155), high-salt-inducible genes accounting for 54% (133), and cold-inducible genes accounted for 10% (25), indicating significant crosstalk between ABA response and abiotic stress signaling pathways. Furthermore, ABA application significantly increased the activities of PAL and POD enzymes in tomato callus, highlighting its crucial role in suppressing the induction of fruit skin damage, which is of great importance for post-harvest preservation and maintenance of visual quality (Tao et al., 2016). Thus, the use of ABA treatment is of great importance to increase the content of health compounds and help plants adapt to stress conditions as part of pre-harvest regulatory measures.

#### Methyl jasmonate

Methyl jasmonate (MeJA), derived from jasmonic acid as a volatile methyl ester, plays a crucial role in plant signaling networks with diverse functions, including post-harvest preservation, quality improvement, stress response and induction of plant immunity (Wang et al., 2021b, 2022a; Kamakshi et al., 2023). MeJA treatment for 3 days before harvest has demonstrated efficacy in promoting the synthesis of secondary metabolites in the leaves of Chinese cabbage and leek, such as total flavonoids, phenolic compounds, vitamin C, soluble sugars and soluble proteins, thereby improving the nutritional quality. In addition, MeJA induction enhances the production of enzymatic pyruvate and aromatic compounds, contributing to improve crop flavor (Baek et al., 2021; Wang et al., 2021a). MeJA also significantly enhances the antioxidant and disease resistance of crops, thereby regulating the post-harvest quality. Spraying an appropriate concentration of MeJA on Chinese cabbage leaves (0.5 mM MeJA, 3 days before harvest), Chinese chive (0.5 mM MeJA, 3 days before harvest) and broccoli (0.04 mmol MeJA for 10 times from germination stage to harvest) elevate antioxidant activity, attributed to increased total GSLs content, DPPH free radical scavenging ability, oxygen radical absorbance capacity (ORAC), FRAP and ABTS (Baek et al., 2021; Wang et al., 2021a, 2022a). The increase in GSLs content in broccoli was attributed to the significant increase in the expression of key genes (CYP83A1, SUR1, UGTB1 and SOT18) involved in the biosynthesis of aliphatic GSLs (AGS) in plants after exogenous application of MeJA (Wang et al., 2022a). Additionally, MeJA exhibits strong antifungal activity, inhibiting spore germination, production and germ tube elongation, thus playing an important role in plant disease resistance (Wang et al., 2021b). MeJA induced resistance reduces symptoms of tomato early blight by 60% after a 48h application (Kamakshi et al., 2023). Furthermore, MeJA positively influences the induction of anthracnose resistance in plants, enhancing defense-related enzyme activities such as chitinase and PAL, and promoting the expression of defense-related genes, including NPR1, EDS1, ACO1, NP24, and LoxD, thereby triggering plant immunity (Glowacz et al., 2017; Kamakshi et al., 2023). Therefore, the application of MeJA in pre-harvest stage proves beneficial in improving crop, prolonging storage period and inhibiting diseases.

#### Gibberellin

Gibberellin (GA_3_), a naturally occurring plant growth regulator, plays an important physiological role in plant growth and helps plants cope with abiotic stress. Treatment with 10-6 M GA_3_ for 21 days before harvest resulted in a significant increase in lettuce and sesame yields by 48.5% and 41.4%, respectively (Miceli et al., 2019). Exogenously applied GA_3_ also significantly enhanced seed production, with 200 mg·L^-1^ optimizing the seed yield in cauliflower (Prodhan et al., 2022) and radish (Bhattarai, 2017). Moreover, exogenously applied GA_3_ prolongs the shelf life and enhances the nutritional value in plants, recognized for its non-toxic, biodegradable, and residue-free nature, which plays a vital economic role (Yu et al., 2006; Martínez‐Romero et al., 2008; Ding et al., 2015; Camara et al., 2018). Additionally, exogenous GA_3_ reduces the activity of enzymes responsible for the degradation of cell structure, thus preserving the integrity (Miceli et al., 2019). The application of GA_3_ before crop harvest has attracted much attention due to its safety and high efficiency.

The pre-harvest supplementation of plant growth regulators is beneficial for enhancing the taste, color, aroma, and other quality attributes of vegetables, thereby enhancing the market competitiveness of the products. Moreover, it can bolster plant resistance to environmental stressors, fortify drought and disease resistance, thus reducing reliance on chemical pesticides and aligning with the principles of sustainable agricultural development. However, challenges exist in practical applications, such as issues related to poor adaptability and instability of certain growth regulators, diminishing their efficacy. Additionally, the application of plant growth regulators necessitates careful consideration of dosage, as excessive usage may compromise the original flavor of vegetables. Furthermore, it’s important to acknowledge that some growth regulators may persist in vegetables, posing potential risks to human health.

Therefore, future advancements in pre-harvest plant growth regulator application should focus on several key areas. Firstly, there’s a need to prioritize the safety and environmental compatibility of selected growth regulators. Research should aim to ensure vegetable quality while minimizing residual levels of growth regulators to uphold food safety and environmental sustainability. Exploring safer and more efficient growth regulators, such as synthesizing plant hormone compounds or developing natural extracts, can address this need and meet agricultural production requirements. Secondly, further investigation into the regulatory mechanisms of growth regulators on plant growth and development is essential. This includes studying signal transduction pathways, gene expression regulation, and other aspects to enhance the efficacy of regulator applications. Lastly, the development of intelligent growth regulator application technologies is crucial. These technologies can enable precise regulation of vegetable growth processes, thereby enhancing production efficiency and quality.

## Perspective

In controlled environment agriculture, regulating the growth conditions of fruits and vegetables before harvest can have numerous benefits. Adjusting the nutrient solution, implementing CL, light supplementation, and exogenous hormone application can effectively enhance the nutritional quality of crops, optimize their color, aroma, and taste, delay harvest senescence and physiological disorders, induce post-harvest disease resistance, and extend storage tolerance and shelf life. Pre-harvest regulation offers advantages such as a short application time, significant effects, lower input compared to application during the growth period, and being non-toxic and non-polluting. Consequently, it has garnered attention from researchers and holds great potential for further research and production. Previously, we summarized the effect of single pre-harvest practice on horticultural crops’ qualities. Moreover, a combination of two preharvest practices resulted in greater improvement in crop quality compared to using a single preharvest practice alone (Liang et al., 2022). For example, nitrogen depletion and short-term continuous LED light treatment have been shown to effectively enhance nutrient quality. However, treating plants with nitrogen deficiency for 5 to 7 days led to reduced yield. In a recent study, Yang et al. (2023) demonstrated a feasible strategy by combining nitrogen deficiency treatment with continuous LED light for 3 days, resulting in significant improvements in various qualities, including soluble sugar, soluble protein, polyphenols, flavonoids, and phenolic acids. Notably, this treatment approach had the additional advantage of conserving energy without compromising yield. Therefore, in agricultural practice, the synthetic strategy with several pre-harvest practices combined together may have great potential to be explored in the future.

**Figure 1:**
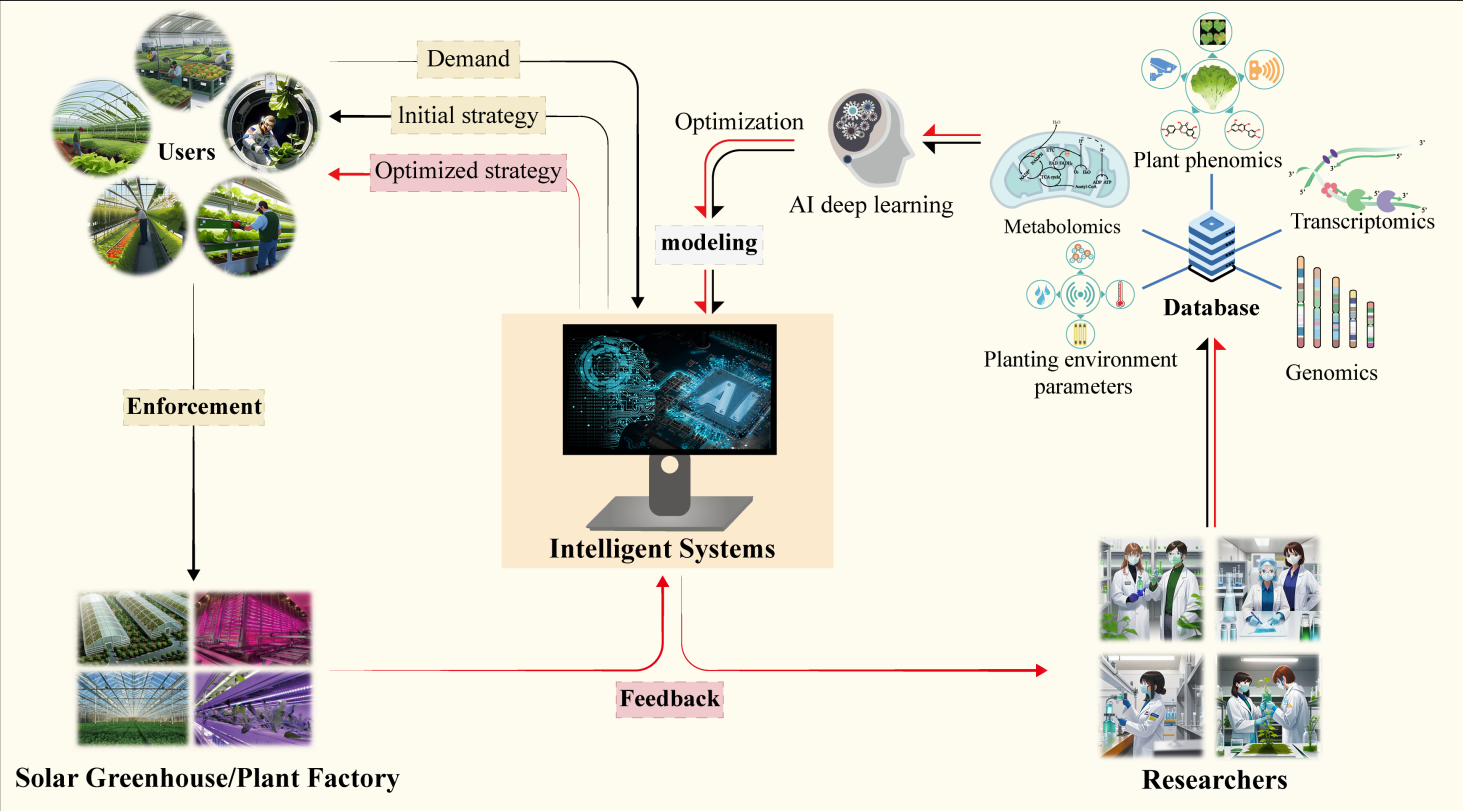
A framework for producing high quality horticultural crop by a intelligent pre-harvest management system.

In future research and application promotion, different combinations and doses of pre-harvest regulation measures can be applied according to the use of target plants. It is also worth noting that because the pre-harvest regulation is dependent on a line of technology carried out in the facility and the regulation measurement suitable for different plants varies greatly, it may be possible to use intelligent plant factories and artificial intelligence in the future. Combining the environmental conditions that different varieties of crops already have in production and the goals that need to be improved to output the most appropriate pre-harvest regulation measures to form a highly intelligent and efficient pre-harvest regulation production system (**Figure 1**).

In the future, plant factories will be equipped with advanced sensor networks to monitor crop growth status, nutritional requirements and environmental conditions in real-time. Based on these data, the intelligent control system will adjust the application amount and timing of measures such as nutrient stress, supplementation of trace elements and regulation of light environment, to realize precise fertilization and environmental regulation. In addition, with the continuous development of intelligent technology, there will be a more intelligent pre-harvest control system, which can adjust parameters timely according to the growth status and environmental conditions of horticultural crops, and realize the dynamic adjustment of pre-harvest control. This will help to optimize the allocation of resources, improve production efficiency, and reduce energy consumption (**Figure 1**).

In summary, the short-term pre-harvest biofortification measures in the future intelligent plant factory will become an important agricultural management technology. Through the guidance of technological innovation and sustainable development concepts, the intelligent, efficient and environmentally friendly agricultural production will be realized.

## Author contributions

XZ: Conceptualization, Writing – original draft, Writing – review & editing. JP: Writing – original draft, Writing – review & editing. LZ: Investigation, Writing – review & editing. XY: Investigation, Methodology, Writing – review & editing. YQ: Resources, Software, Writing – review & editing. CC: Software, Validation, Writing – review & editing. JH: Software, Validation, Writing – review & editing. TH: Resources, Writing – review & editing. YL: Software, Validation, Writing – review & editing. ZL: Resources, Writing – review & editing. MT: Funding acquisition, Writing – review & editing. FL: Project administration, Supervision, Writing – review & editing. ZW: Conceptualization, Project administration, Writing – review & editing.
